# Editorial Expression of Concern: Experimental orthotopic transplantation of a tissue-engineered oesophagus in rats

**DOI:** 10.1038/ncomms13310

**Published:** 2016-10-14

**Authors:** Sebastian Sjöqvist, Philipp Jungebluth, Mei Ling Lim, Johannes C. Haag, Ylva Gustafsson, Greg Lemon, Silvia Baiguera, Miguel Angel Burguillos, Costantino Del Gaudio, Antonio Beltran Rodriguez, Alexander Sotnichenko, Karolina Kublickiene, Henrik Ullman, Heike Kielstein, Peter Damberg, Alessandra Bianco, Rainer Heuchel, Ying Zhao, Domenico Ribatti, Cristián Ibarra, Bertrand Joseph, Doris A. Taylor, Paolo Macchiarini

Nature Communications 5: Article number: 4562; DOI: 10.1038/ncomms4562(2014); Published: 04 15 2014; Updated: 10 14 2016.

*Nature Communications* is publishing an editorial expression of concern on the manuscript ‘Experimental orthotopic transplantation of a tissue-engineered oesophagus in rats’ from Sjöqvist *et al.* to alert our readership to concerns regarding the integrity of the study. An investigation related to this research has been conducted by the Expert Group for Misconduct in Research at the Swedish Central Ethical Review Board on behalf of Karolinska Institutet. A statement on behalf of the Expert Group summarizing the results of this investigation ( http://www.epn.se/media/2374/o-1-2016-statement-expert-group-for-misconduct-in-research-160906-eng.pdf) raises concerns regarding the extent to which the data presented in this Article accurately report and are fully representative of the results of the experiments that were carried out. Concerns have been raised regarding the *in vitro* characterization of the oesophageal scaffold, *in vivo* imaging of the transplanted oesophageal scaffold and the degree to which its transplantation into animals was successful. We are currently following our established process to investigate these issues further.

P.M., M.A.B., H.K., P.D., B.J. and D.A.T. agree, whereas S.S., P.J. and M.L.L. disagree with this notice. The other authors could either not be reached or did not provide a clear response.

